# A conversation with Dr. Kevin Magnaye, Data Scientist, Caris Life Sciences

**DOI:** 10.1017/cts.2024.23

**Published:** 2024-02-23

**Authors:** Clinical Research Forum

**Affiliations:** Clinical Research Forum, Washington, DC, USA

## Top 10 Clinical Research Achievement Awards Q & A

This article is part of a series of interviews with recipients of Clinical Research Forum’s Top 10 Clinical Research Achievement Awards. This article is with Dr Kevin Magnaye, Data Scientist, Caris Life Sciences. While a graduate student with Dr Carole Ober at the University of Chicago, Dr Magnaye explored genetic and epigenetic mechanisms underlying maternal transmission of asthma risk. Currently, Dr Magnaye, as part of the Next Generation Profiling team at Caris Life Sciences, helps to develop molecular signatures to advance precision medicine efforts in oncology. He received a 2023 Top 10 Clinical Research Achievement Award for *DNA methylation signatures in airway cells from adult children of asthmatic mothers reflect subtypes of severe asthma. The interview has been edited for length and clarity.*

### When Did You First Become Interested in Pursuing a Career in Science?

It all stems from when I was an undergraduate. I’ve always had an interest in understanding why we are the way we are, and at the University of Washington, Seattle, I was fortunate to have an amazing network of mentors who cultivated my growth as a scientist. They helped me think about ways to connect our biology to what we do and how we act, and I ended up earning degrees in both molecular biology and anthropology.

### Did that Lay the Foundation for Becoming a Clinical Researcher?

Yes. After college, I went to the University of Chicago for my doctoral degree in human genetics and that’s where I was introduced to clinical research. Initially, I was interested in understanding the fundamental processes of gene regulation, but then I started to learn more about the potential for genetic technologies to investigate our genomes and how those findings could have clinical applications. I also began engaging with other scientists and clinicians about their work – from drug target discovery using genomic data to interventions and clinical trials – and that motivated me to explore clinical questions with my own research. Of course, my rapid development as a clinical researcher is attributed to my mentor, Dr Carole Ober, who directed me through my graduate school journey and with the award-winning study. Personally, I’m grateful for my diverse academic experiences because they helped me see connections and get a more holistic view of clinical research.

### What Did Your Award-Winning Study Show?

One of the most widely recognized risk factors for asthma is having a mother with asthma; however, very little is known about the biological mechanisms underlying this association. This study investigated epigenetic changes in lung cells from adult asthma patients with and without an asthmatic mother, and we found an epigenetic profile that differed between these two groups. This profile was associated with a hard-to-treat form of severe asthma only in patients with an asthmatic mother. It was also associated with an aberrant transcriptional signature in lung cells that reflected an apparent response to viral and bacterial pathogens. Overall, our study suggests that the early life environment of a mother with asthma alters the epigenetic landscape in her fetus that persists through the lifespan.

### How Did Team Science Contribute to the Success of the Study?

Team science played a crucial role in this achievement. It was a collaborative effort that involved hundreds of people nationwide, including researchers, clinicians, technicians, and numerous people who assisted with sequencing technologies and computational approaches. I also want to recognize the participants who were so generous in contributing their samples. About 10% of the general population has asthma, but the most affected are those from historically underrepresented backgrounds such as individuals with African ancestry. Our study included two-thirds African-American subjects and having this representative population was key for us to translate these findings across groups.

### How Will These Findings Advance the Field of Asthma Research?

First, we identified a relationship between the early life environment of a mother with asthma that associates with an epigenetic trajectory to asthma later in life. Second, our findings could also be used to develop novel therapeutic targets for severe asthma. For instance, severe asthmatics with a T2-high endotype can be managed with corticosteroids and T2 cytokine-targeting therapies, but there aren’t effective therapies that are specifically targeted for people with T2-low severe asthma.

### Is This Research Continuing?

While I was a postdoctoral fellow at the University of California San Francisco, I conducted follow-up studies to specifically explore what kind of exposures in early life might mediate this epigenetic trajectory to asthma. I investigated the role of the early life microbiome and its relationship to epigenetics throughout the lifespan. The study that won the award implicated various transcriptional pathways and machinery related to the response to bacterial and viral pathogens, and so a potential signal early in life that may be promoting this trajectory could be early life exposure to pathogenic microbes in the infant’s developing microbiome. It’s also well known, for example, that mode of birth is one of the most significant factors that is associated with alterations in an infant’s gut microbiome and I had investigated these potential mechanisms among others. This work is soon to be published. Stay tuned!

### What Advice Do You Have for Someone who’s Thinking About a Career in Clinical Research?

It’s early in my own career but reflecting on the past few years, I can offer two pieces of advice. First, looking back, I wish I had been less fearful of the unknown. Certainly, for any scientist, researcher, or anyone going through graduate school, life is a constant roller coaster, and you’re always wondering if you are pursuing the right question and dedicating your time in the best way. But my advice is to not fear the unknown – because often, it’s the unknown that leads to growth and enrichment. That’s what happened with our study. When I was exploring the data, I started to see these epigenetic marks and at first, we didn’t know if they were biologically meaningful. So, I integrated transcriptomic and other types of molecular and clinical data to get a fuller picture and see if they related to subtypes of the disease. Second, I’d say that it’s important to continuously adapt to our ever-evolving world. You need to keep an open mind and embrace curiosity, always staying willing to pivot and adjust your ideas.

### What Do You Enjoy Doing Outside of the Lab and How Do Your Hobbies Impact Your Work?

When I studied anthropology as an undergraduate, I became immersed in trying to understand different cultures, and I still enjoy doing that by traveling or meeting new people or even just opening my new edition of *National Geographic*. It expands my perspective and has helped me to become a better researcher, a better geneticist, and, most importantly, a better person. For me, exploring different cultures and environments reinforces the global nature of medical research and healthcare, and it allows me to think of my research in a broader, more complete way. It reminds me that there are so many different factors that influence us – whether they are biological, environmental, or socioeconomic – and we need to always keep that in mind. That’s where the best, most meaningful science happens – when we’re thinking holistically.

